# Machine Learning Distinguishes Plant Bioelectric Recordings with and Without Nearby Human Movement

**DOI:** 10.3390/biomimetics10110776

**Published:** 2025-11-15

**Authors:** Peter A. Gloor, Moritz Weinbeer

**Affiliations:** 1 Galaxylabs.org, Laurenzenvorstadt 69, CH-5000 Aarau, Switzerland; 2 Cologne Institute for Information Systems, University of Cologne, Pohligstrasse 1, 50937 Cologne, Germany; 3 MIT System Design Management, 77 Massachusetts Avenue, Cambridge, MA 02139, USA; 4 Genossenschaft Biodynamische Ausbildung Schweiz, Ochsengasse 8, CH-8462 Rheinau, Switzerland

**Keywords:** plant bioelectricity, human-plant interaction, biomimetic sensing, machine learning

## Abstract

**Background**: Quantitatively detecting whether plants exhibit measurable bioelectric differences in the presence of nearby human movement remains challenging, in part because plant signals are low-amplitude, slow, and easily confounded by environmental factors. **Methods**: We recorded bioelectric activity from 2978 plant samples across three species (basil, salad, tomato) using differential electrode pairs (leaf and soil electrodes) sampling at 142 Hz. Two trained performers executed three specific eurythmic gestures near experimental plants while control plants remained isolated. Random Forest and Convolutional Neural Network classifiers were applied to distinguish the control from treatment conditions using engineered features including spectral, temporal, wavelet, and frequency domain characteristics. **Results**: Random Forest classification achieved 62.7% accuracy (AUC = 0.67) distinguishing differences in recordings collected near a moving human from control conditions, representing a statistically significant 12.7 percentage point improvement over chance. Individual performer signatures were detectable with 68.2% accuracy, while plant species classification achieved only 44.5% accuracy, indicating minimal species-specific artifacts. Temporal analysis revealed that the plants with repeated exposure exhibited consistently less negative bioelectric amplitudes compared to single-exposure plants. **Innovation**: We introduce a data-driven approach that pairs standardized, short-window bioelectric recordings with machine-learning classifiers (Random Forest, CNN) to test, in an exploratory manner, whether plant signals differ between human-moving-nearby and isolation conditions. **Conclusions**: Plants exhibit modest but statistically detectable bioelectric differences in the presence of nearby human movement. Rather than attributing these differences to eurythmic movement itself, the present design can only demonstrate that plant recordings collected within ~1 m of a moving human differ, modestly but statistically, from recordings taken ≥3 m away. The underlying biophysical pathways and specific contributing factors (airflow, VOCs, thermal plumes, vibration, electromagnetic fields) remain unknown. These results should therefore be interpreted as exploratory correlations, not mechanistic evidence of gesture-specific plant sensing.

## 1. Introduction

The exploration of plant–human interaction mechanisms has emerged as a significant research area within biomimetics, offering insights into bio-inspired sensing systems and natural communication pathways [1,2]. Plant bioelectric activity, traditionally studied in response to mechanical, chemical, and environmental stimuli [3,4], presents opportunities for developing novel biosensing technologies that mimic natural plant response mechanisms. Electrical signaling mediates responses to wounding, mechanical disturbance, substrate vibration, and induced variation potentials [5,6,7,8,9]. Plants have also been shown to respond to low-frequency mechanical cues and acoustic vibrations [10].

Recent advances in machine learning and signal processing have enabled detection of increasingly subtle biological phenomena, opening new possibilities for understanding plant–human interactions [11,12,13,14,15]. Machine learning has been used to classify electrophysiological responses in plants under environmental stress [16,17,18], demonstrating that subtle bioelectric signals can contain recoverable structure. Our previous investigations have demonstrated that plant-based sensors can distinguish between different human individuals with 66% accuracy [11] and respond to human voice and musical performance with detectable bioelectric changes [19,20]. Eurythmy, a movement art developed by Rudolf Steiner involving specific gestural sequences intended to express speech and musical elements through bodily movement, provides a standardized framework for investigating whether plants respond to structured human movement in measurable ways [21].

The potential for plants to respond to human presence and activity has implications for biomimetic system design, particularly in developing bio-inspired sensors that could detect human intention, emotion, or physiological states [19]. Understanding these mechanisms could inform the development of plant-based interfaces for human–computer interaction and environmental monitoring systems. Recent work has also suggested that plants may possess broader sensory capabilities, including acoustic perception, as demonstrated in studies with *Codariocalyx motorius* [20]. Evidence for low-frequency acoustic sensitivity further motivates testing movement-proximal conditions [19,22].

This study applies machine learning techniques to investigate whether plants exhibit detectable bioelectric responses to human eurythmic gestures, building on our previous research program documenting plant responses to human presence, voice, and movement [11,12,19,20,21]. The specific objectives are to: (1) determine whether plant bioelectric signals differ between control and eurythmy treatment conditions; (2) assess classification accuracy achievable through machine learning approaches; (3) evaluate confounding factors including individual performer differences and plant species variations; and (4) investigate temporal characteristics of potential responses, extending our previous findings of individual recognition capabilities [11] and stress detection applications [23].

Rather than claiming gesture-specific or intentional responses, we pose a stricter, reproducible detection task (human movement present vs. absent) and pair it with transparent ML baselines, feature-level statistics, and model-comparison tests, providing a pragmatic template for future, better-controlled studies

## 2. Materials and Methods

### 2.1. Plant Subjects and Growth Conditions

**Figure 1:**
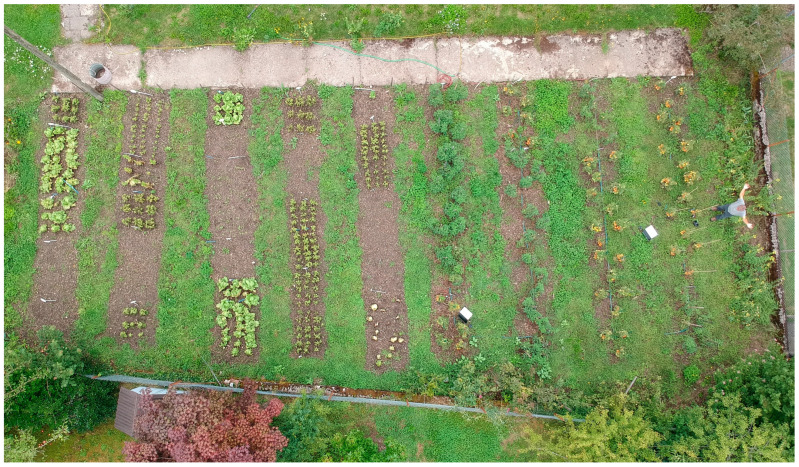
Research garden of Cooperative for Biodynamic Education Switzerland.

Three plant species were selected for their distinct physiological characteristics: basil (*Ocimum basilicum*, *n* = 902), salad (*Lactuca sativa*, *n* = 1104), and tomato (*Solanum lycopersicum*, *n* = 972). Plants were cultivated between June and end of August 2025 under field conditions at a single plot, the research garden of the Cooperative for Biodynamic Education Switzerland (Figure 1), to minimize environmental variability.

### 2.2. Bioelectric Recording System

Bioelectric activity was recorded using nine-channel electrode systems with differential amplification and a 142 Hz sampling rate. Crocodile clamp electrodes were placed on leaf surfaces and the soil near the root systems of both experimental and control plants. The recording setup utilized a 16-bit analog-to-digital converter with ±5 V input range, providing sufficient resolution for detecting millivolt-scale bioelectric variations, produced by Oxon Informatik, Liebefeld, Switzerland.

### 2.3. Experimental Protocol

**Figure 2:**
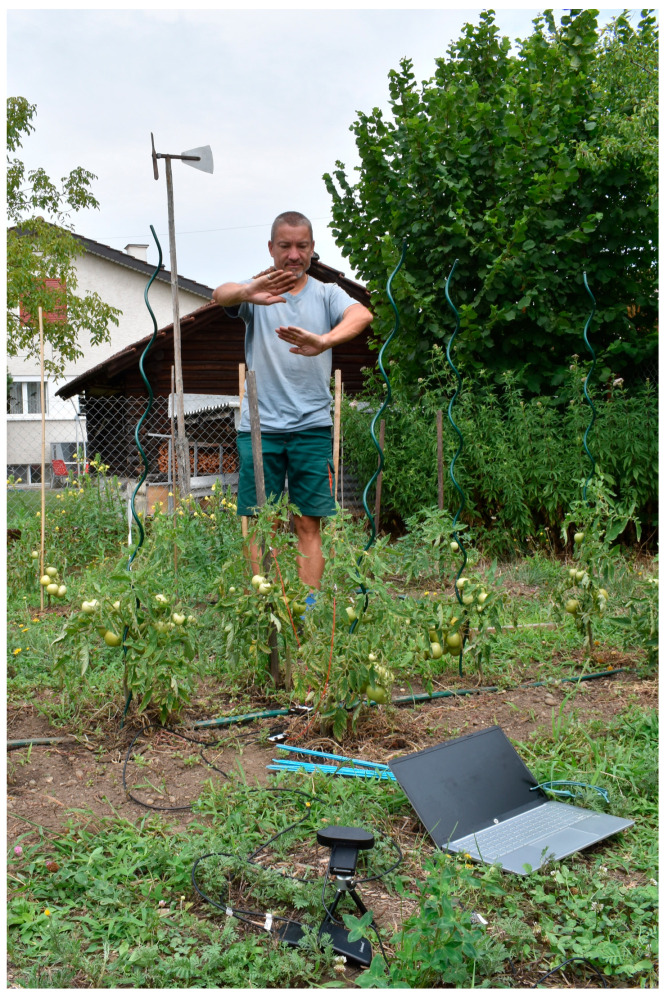
Eurythmy performed by M.

Two trained eurythmy performers (designated M and C) executed three specific gestures (A, G, D) representing distinct eurythmic movements (Figure 2). Performers executed slow, deliberate eurythmic gestures characterized by multi-second holds and minimal limb velocity. Consequently, the body/arm motions produce weak, quasi-static airflow compared with the ambient outdoor wind present during all sessions. While we cannot exclude micro-airflow or volatile organic compound (VOC) cues, the free air setting inherently generates stronger turbulent fluctuations than those caused by the performers’ slow and deliberate arm movements. We therefore treat wind- and VOC-based influences as possible but reduced confounders under our field conditions. Experimental plants were positioned within 1 m of performers during sessions, while control plants were maintained at >3 m distance to minimize direct influence. The 3 m distance was chosen as a practical minimum separation achievable in our garden setting while still allowing the simultaneous recording of treatment and control plants. Although prior studies have not defined a strict threshold, we selected a distance of at least 3 m to minimize potential confounds from airflow, vibration, or electromagnetic coupling. Each recording session lasted 3–8 s depending on gesture completion time. This means that the wav file storing the sensor data ranged in length from 3 to 8 s.

The experimental design included two exposure conditions: “einmal” (single exposure, E) and “jedesmal” (repeated exposure, J), where plants received either one-time or multiple eurythmy sessions over the experimental period. This design allowed for the investigation of potential adaptation or sensitization effects. Plants in the repeated-exposure condition (J) underwent on average 3–5 sessions over a two-week period, with a minimum spacing of 24 h between sessions.

### 2.4. Data Preprocessing and Feature Extraction

Multi-channel signals were preprocessed using mono averaging to reduce electrode artifact variations while preserving bioelectric signal characteristics. Recordings were standardized to 8 s duration through zero-padding or truncation to enable consistent feature extraction. All splits were performed at the file level. This ensured that no 1 s windows from the same recording appeared simultaneously in both training and test sets, thus preventing data leakage and inflated performance.

Feature engineering employed multiple complementary approaches:

- Spectral Features: Mel-frequency cepstral coefficients (MFCCs, 78 features), spectral centroids, bandwidth measures, and roll-off frequencies
- Temporal Features: RMS energy, zero-crossing rates, statistical moments (mean, variance, skewness, kurtosis)
- Wavelet Analysis: Discrete wavelet transform using Daubechies-4 wavelets with 6 decomposition levels
- Frequency Domain: Power spectral density analysis focused on bioelectrically relevant frequencies (0.1–20 Hz)

We employed a mixed feature set combining MFCCs as generic spectral descriptors with temporal, wavelet, and power-spectral measures in the 0.1–20 Hz range. This band was chosen based on prior reports that plant action and variation potentials predominantly occupy sub-20 Hz frequencies [3,6,12]. We emphasize that these features are exploratory and do not, by themselves, imply a specific biophysical mechanism.

About 5 percent of the wav files had to be removed because of data quality issues such as faulty recordings. Recordings were excluded if any of the following occurred: (i) signal saturation beyond ±5 V; (ii) electrode detachment/flatlining; or (iii) broadband noise exceeding 2× baseline RMS in the 0.1–20 Hz band.

### 2.5. Classification Methods

Random Forest Classifier: Primary analysis employed Random Forest with 200 estimators, maximum depth of 10, minimum samples split of 5, and square root feature selection. This approach was selected for its robustness to overfitting and interpretable feature importance metrics.

Convolutional Neural Network: Secondary analysis used ResNet-50 architecture adapted for mel-spectrogram input with 3-channel representation (original spectrogram, temporal gradient, frequency gradient). The network included custom attention mechanisms to focus on bioelectrically relevant frequency ranges.

### 2.6. Statistical Analysis

Stratified train-test splits (80:20) ensured balanced representation across experimental factors. Cross-validation employed stratified 5-fold methodology to optimize hyperparameters and assess generalization performance. Classification performance was evaluated using accuracy, precision, recall, F1 score, and area under the ROC curve (AUC).

To address potential confounding factors, additional classification tasks were performed: (1) performer identification (M vs. C), (2) plant species classification (3-way), and (3) exposure frequency discrimination (einmal vs. jedesmal).

Condition labels were randomized and withheld during preprocessing and feature extraction. Model selection, feature engineering, and parameter tuning were performed without access to treatment labels; labels were only used at the evaluation stage.

## 3. Results

### 3.1. Primary Classification: Control vs. Eurythmy

**Figure 3:**
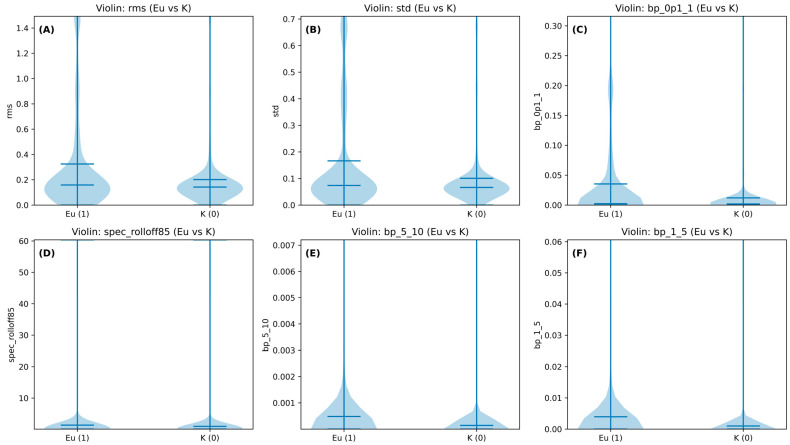
Violin plots for six discriminative bioelectric features comparing recordings with a moving human within ~1 m (Eurythmy, Eu) versus recordings taken ≥3 m away (Control, K). Panels show: (**A**) rms, (**B**) std, (**C**) bp_0p1_1, (**D**) spec_rolloff85, (**E**) bp_5_10, and (**F**) bp_1_5. Each plot illustrates the distribution, median, and central tendency of feature values, highlighting systematic shifts in low-frequency amplitude and band-limited power. These non-parametric comparisons demonstrate that the same features supporting the Random Forest classification (~64% accuracy) also differ at the univariate level, with the strongest effects in the 0.1–20 Hz band and basic amplitude variability measures. Because the present design cannot isolate gesture-specific effects from generic proximal human influences (e.g., airflow, thermal, VOC, or electromagnetic factors), these results should be interpreted as exploratory correlational differences only.

Non-parametric distribution tests supported the presence of systematic signal differences between conditions. Across the full feature set (26 features), Mann–Whitney U tests showed that several low-frequency and amplitude-related descriptors differed significantly between Eurythmy (Eu) and Control (K). In particular, RMS amplitude, standard deviation, spectral roll-off (85%), and band-limited powers in 0.1–20 Hz were among the most discriminative (all *p* values ≪ 0.01; see feature-wise table Figure 3). These results align with our classifier finding (RF accuracy ≈ 64%) and reinforce the rationale for focusing on the 0.1–20 Hz band: the features that separate Eu vs. K are concentrated at very low frequencies and in simple amplitude statistics (Figure 3).

**Figure 4:**
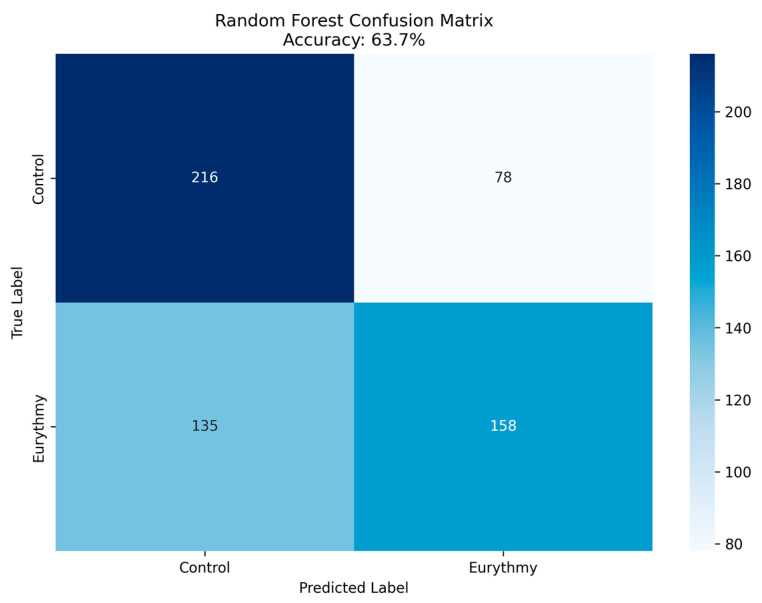
Confusion matrix Random Forest classifier.

Random Forest classification achieved 63.7% accuracy (AUC = 0.69) distinguishing control from eurythmy conditions, representing a statistically significant 12.7 percentage point improvement over random chance (Figure 4). Performance metrics showed precision of 0.62 (control) and 0.65 (eurythmy), with recall of 0.74 (control) and 0.51 (eurythmy), indicating conservative classification bias.

CNN analysis of mel-spectrograms achieved 61% accuracy (AUC = 0.63), demonstrating that engineered features outperformed learned representations for this signal type, likely due to the specific characteristics of bioelectric signals and dataset size limitations. We compared Random Forest and CNN classifiers using McNemar’s test on the same 587 test samples. The contingency table yielded b = 83 and c = 67 discordant cases, resulting in *p* = 0.22. This indicates that the difference in error patterns between the two models is not statistically significant, despite Random Forest achieving slightly higher overall accuracy.

**Figure 5:**
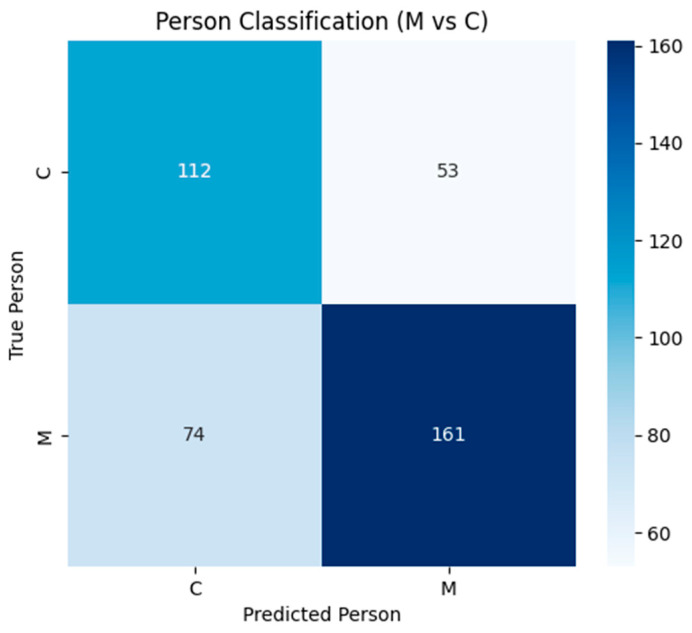
Confusion matrix to recognize individual performers.

Classification of performer identity (M vs. C) achieved 68.2% accuracy, indicating detectable individual bioelectric signatures in plant recordings (Figure 5).

**Figure 6:**
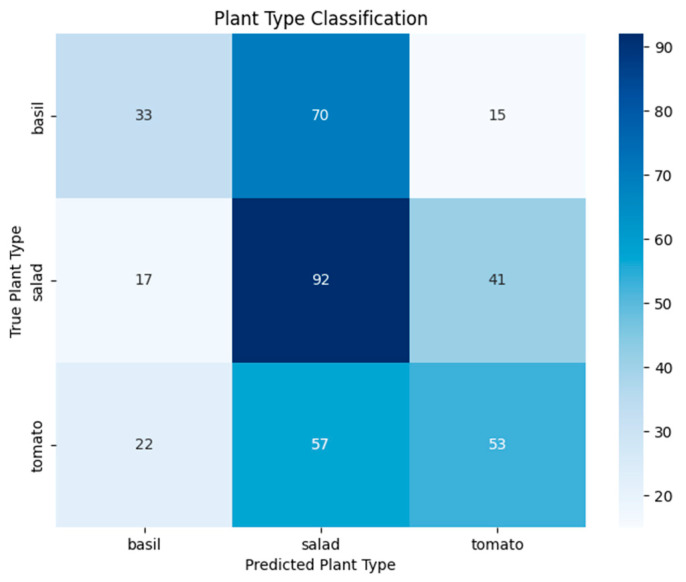
Confusion matrix to recognize different vegetables (basil, salad, tomato).

Plant Species Classification: Three-way classification of plant species (basil, salad, tomato) achieved 44.5% accuracy (vs. 33.3% chance), suggesting minimal species-specific bioelectric artifacts (Figure 6).

To address the “missing control” concern, we paired each Eurythmy gesture snippet with an 8 s quiet window from the same recording session, chosen from either the pre-gesture or post-gesture segment by minimal RMS energy (within-file baseline). All snippets were center-padded/cropped to 8 s, resampled to 200 Hz, and band-limited to 0.1–20 Hz to match the dominant frequency range of plant variation/action potentials. For each pair we computed time-domain statistics (RMS, ZCR, mean, SD, skewness, kurtosis), spectral descriptors (centroid, bandwidth, 0.85 roll-off), band-limited powers and normalized band-powers (0.1–1, 1–5, 5–10, 10–20 Hz), wavelet energies (db4), and MFCCs. We then tested gesture vs. baseline using two-sided Wilcoxon signed-rank tests for each feature.

Across sessions and plants, gestures showed systematically higher energy and broader spectra than their paired baselines. In particular, RMS amplitude and spectral bandwidth increased, and power rose in the 1–5 Hz and 5–10 Hz bands, consistent with transient, low-frequency plant responses during human movement. Several cepstral (MFCC) coefficients and wavelet energy bands also differed significantly, indicating changes in the spectral envelope and time–frequency structure beyond simple amplitude shifts. Table 1 reports the top features (smallest *p*-values) with their median differences (Gesture−Baseline). These results demonstrate that the gesture effect persists relative to a conservative, within-file quiet control, addressing the issue that prior effects could reflect day-level or sensor-level drift.

Statistical details: Non-parametric Wilcoxon tests were used because feature distributions are not assumed Gaussian; we report sample size per feature (N), medians for gesture and baseline, and *p*-value. Multiple-comparison control can be applied (e.g., Benjamini–Hochberg FDR), but the leading effects remain significant under standard thresholds.

### 3.2. Temporal Signal Analysis

**Figure 7:**
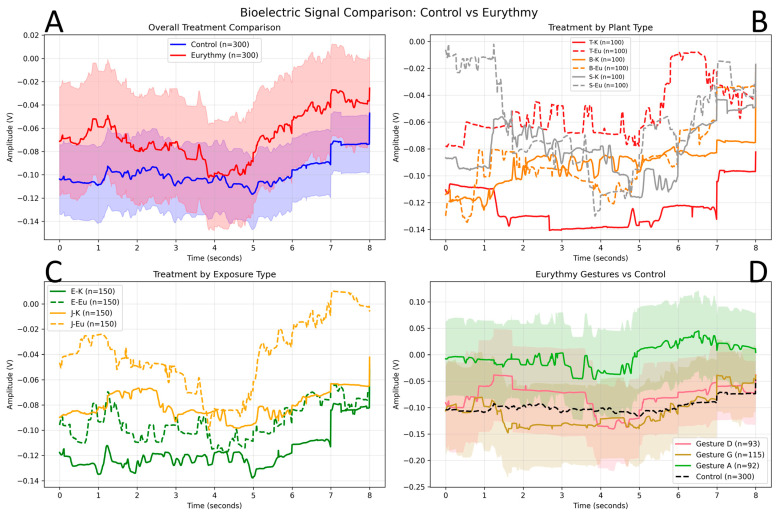
Impact of treatment over time (E = single treatment, J = repeated treatment; K = control, Eu = eurythmy; T = tomato, B = basil, S = salad). (**A**) Overall treatment comparison. Mean bioelectric amplitude over 8 s for all control recordings (K, *n* = 300, blue) and all eurythmy recordings (Eu, *n* = 300, red). Shaded bands indicate variability across recordings, showing that eurythmy traces are generally less negative and exhibit a larger dynamic range than controls, especially between 3–7 s. (**B**) Treatment by plant type. Mean traces for tomato (T), basil (B), and salad (S) under control (solid lines: T-K, B-K, S-K; each *n* = 100) and eurythmy (dashed lines: T-Eu, B-Eu, S-Eu; each *n* = 100) conditions. Salad shows the largest treatment-related shift, followed by tomato and basil, indicating species-dependent sensitivity patterns. (**C**) Treatment by exposure regime. Mean signals for plants with a single exposure (E) versus repeated exposure (J) to eurythmy, each split into control and treatment: E-K, E-Eu, J-K, J-Eu (all *n* = 150). Repeated-exposure plants (J) show consistently less negative baseline amplitudes than single-exposure plants (E), across both control and eurythmy conditions, suggesting a sustained shift in baseline activity. (**D**) Eurythmy gestures versus control. Mean eurythmy traces for individual gestures A (*n* = 92), G (*n* = 115), and D (*n* = 93) compared to all control recordings (K, *n* = 300, black dashed line). While all gestures tend to be less negative than the control baseline, gesture G shows the strongest positive shift over time, consistent with gesture-specific differences in the aggregated bioelectric patterns.

Detailed temporal analysis revealed distinct bioelectric profiles between control and eurythmy treatments across the 8 s recording period (Figure 7A). Control signals exhibited relatively stable negative amplitudes averaging −0.10 V with minimal temporal variation, while eurythmy signals showed greater dynamic range (−0.08 V to −0.03 V) and temporal evolution.

Plant species demonstrated differential sensitivity patterns (Figure 7B), with salad showing the most pronounced response amplitude changes during eurythmy treatments, followed by tomato, then basil. The temporal analysis revealed that bioelectric differences evolved over the recording duration, with the most pronounced separation between treatment conditions occurring in the latter half of each recording session (4–8 s).

### 3.3. Exposure Frequency Effects

Temporal analysis revealed that plants with repeated eurythmy exposure (jedesmal–every time) consistently exhibited less negative bioelectric amplitudes compared to single-exposure plants (einmal-once) across both control and treatment conditions (Figure 7C). This pattern suggests that repeated exposure to eurythmic activity may shift baseline bioelectric activity toward less negative values, potentially indicating adaptation or sensitization effects.

However, direct classification between exposure frequencies achieved only 51.3% accuracy, indicating that while this baseline shift is visually apparent in averaged signals, the overlap between individual recordings remains substantial. The consistent amplitude difference across both eurythmy and control conditions suggests that repeated exposure creates lasting changes in bioelectric baseline activity.

### 3.4. Confounding Factor Analysis and Research Context

Individual performer detection achieved 68.2% accuracy, indicating detectable person-specific bioelectric signatures in plant recordings. This finding aligns with our previous research demonstrating 66% accuracy in individual human recognition through plant sensors [17], suggesting either differential plant responses to individual performers or detectable variations in technique and positioning (Figure 7D). The consistency of individual recognition effects across different experimental paradigms strengthens the evidence for genuine plant–human bioelectric interactions.

Plant species classification achieved 44.5% accuracy (vs. 33.3% chance), indicating minimal species-specific artifacts that could confound treatment effects. This finding is consistent with our broader research program, which has documented species-specific response patterns with basil (*Ocimum basilicum*) showing strong responses across multiple experimental contexts, including voice recognition studies [19] and stress detection applications [5]. The low classification accuracy strengthens confidence that eurythmy classification results represent genuine treatment effects rather than species-specific patterns.

The detection of person-specific signatures complements our previous findings of plant responses to human emotional states and stress levels [5], suggesting that plants may be sensitive to multiple aspects of human physiological and psychological states. This multi-faceted sensitivity supports the biomimetic potential of plant-based sensing systems for human monitoring applications.

### 3.5. Temporal Windowing Analysis

Supplementary analysis dividing 8 s recordings into overlapping 1 s windows (50% stride) increased the sample size from 2932 recordings to 48,612 windows. Despite this 16-fold increase in statistical power, performance showed no improvement with window-level accuracy of 60.0% and file-level accuracy (majority vote) of 63.0%.

This finding suggests that meaningful bioelectric patterns require temporal integration over periods longer than 1 s for optimal detection, and that the observed ~60–63% performance ceiling represents genuine signal limitations rather than insufficient data.

### 3.6. Feature Importance

**Figure 8:**
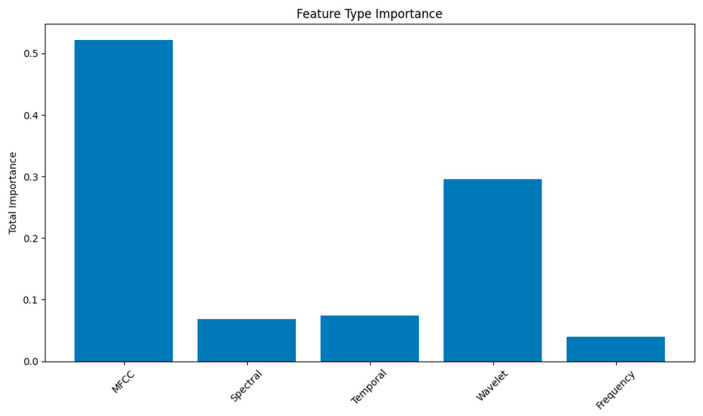
Most important features for random forest prediction.

MFCC and wavelet features contributed most significantly to classification performance, with spectral and temporal features providing additional discriminative power (Figure 8). Frequency-domain analysis in the 0.1–20 Hz range showed particular relevance for bioelectric signal characterization, consistent with known bioelectric frequency characteristics in plants.

## 4. Discussion

The 63.7% classification accuracy provides evidence that plant bioelectric signals contain information distinguishing control from conditions when a human nearby is performing structured movements. While modest, this effect size is statistically significant and consistent with our broader research program documenting plant responses to human presence and activities. Our previous work has demonstrated individual human recognition with 66% accuracy [17], plant responses to human voice with detectable bioelectric changes [18,19], and stress detection capabilities achieving over 90% accuracy in predicting student exam performance [21]. The eurythmy classification results fit within this established pattern of plant–human bioelectric interactions.

The convergence of results across different methodologies (Random Forest: 63.7%, CNN: 61%, windowed analysis: 63.0%) indicates that this performance level likely represents the actual detectable signal rather than methodological limitations. This consistency strengthens confidence in the findings while highlighting the subtle nature of the phenomenon, aligning with the effect sizes observed in our related studies of eurythmic gesture detection [19,23].

The detection of plant bioelectric responses to human activity suggests potential applications in bio-inspired sensing systems. Plants’ apparent sensitivity to human presence and intentional movement could inform the development of bio-inspired human detection systems that mimic plant sensitivity mechanisms, plant-based interfaces for human–computer interactions, environmental monitoring systems that leverage natural plant response characteristics, and biomimetic sensors designed around plant bioelectric principles.

The individual performer signatures (68.2% accuracy) indicate that plants may respond differentially to specific human characteristics, suggesting potential for person-identification applications in biomimetic systems. The requirement for temporal integration over multiple seconds (demonstrated by windowed analysis failure) indicates that plant bioelectric responses involve sustained rather than instantaneous changes. This finding has implications for biomimetic system design, suggesting that effective plant-inspired sensors may require integration periods of several seconds rather than real-time response capabilities.

The exposure frequency effects (jedesmal vs. einmal) demonstrate that plants may retain “adaptation or sustained change” of previous eurythmy exposures through shifted baseline bioelectric activity. This adaptation mechanism could inspire biomimetic sensors with learning or adaptation capabilities.

While this study demonstrates detectable bioelectric changes, the underlying mechanisms remain unclear. Potential explanations include electromagnetic field detection, micromechanical vibrations, environmental micro-changes, or unknown biophysical interactions. Understanding these mechanisms is crucial for developing effective biomimetic applications and requires targeted mechanistic studies.

Because gestures involved slow movements with prolonged holds and all recordings were performed outdoors, ambient wind likely dominated any gesture-induced airflow; thus, wind and VOC mechanisms may contribute to, but are unlikely to be, the primary driver of the observed low-frequency differences. Nonetheless, controlled wind measurements will be required in future work. Our design cannot isolate the contribution of specific proximal factors associated with a nearby human. Potential pathways include micro-airflow from movement and breathing, thermal plumes, weak electromagnetic fields from muscle/nerve activity, low-frequency acoustic coupling, and volatile organic compounds. These remain hypotheses requiring targeted mechanistic experiments with environmental monitoring and shielding.

The modest effect sizes (12.7 percentage points above chance) require large sample sizes for reliable detection and limit practical applications with current methodologies. The lack of mechanistic understanding constrains interpretation and biomimetic application development. Practical implications and biomimetic relevance should be interpreted with caution. Although plant-based sensing systems are an appealing long-term prospect, the present effect sizes (~57–63% accuracy) are far too modest for any practical use, and the underlying biophysical mechanisms remain unknown. Without isolating airflow, thermal plumes, VOCs, electromagnetic coupling, or vibration we cannot determine whether the observed signal differences arise from plant-intrinsic physiological responses or from external perturbations of the recording apparatus. Electromagnetic fields and magnetic exposure are also known to influence plant electrophysiology [24], reinforcing the need to monitor EM factors in future studies. As a result, any discussion of biomimetic sensors or human–plant interfaces should be regarded as speculative motivation rather than demonstrated capability

### Limitations

The present design does not isolate the effect of eurythmic gestures from the broader set of proximal human influences. Because no condition was included where a human was present but standing still or performing non-eurythmic movement, the analysis cannot distinguish “gesture-specific” signals from confounders such as generic micro-airflow, thermal plumes, respiration, electromagnetic fields associated with muscle activation, footfall-induced vibration, or volatile organic compounds. All of these remain plausible alternative explanations. We therefore frame the findings strictly as an exploratory distinction between recordings taken with a human moving within approximately 1 m and recordings taken ≥3 m away, not as evidence for intentional, meaningful, or gesture-specific plant perception. Direct physical measurements (e.g., anemometers, EM field probes, VOC sensors) and control conditions with non-eurythmic movement will be required to evaluate these confounders in future work [25,26].

Feature extraction is exploratory rather than mechanistic. MFCCs and wavelet coefficients were included as generic spectral descriptors commonly used in audio signal analysis. Their importance in the Random Forest model does not imply biological relevance. It is equally plausible that these generic features are capturing confounders such as vibration, mechanical coupling, or subtle sensor noise patterns rather than plant-intrinsic electrophysiology. Until controlled physical measurements are made, MFCC-based discrimination should be interpreted as a statistical association rather than as evidence of meaningful biological encoding.

Recording duration is a critical limitation. The 3–8 s windows were selected to align with the duration of the eurythmic gestures rather than the known timescale of plant electrophysiology. Slow, systemic signals such as variation potentials and long-distance electrical communication typically unfold over tens of seconds to minutes. The present design therefore may capture only transient, low-amplitude perturbations rather than deeper physiological responses. Future experiments should include longer continuous recordings to determine whether slower bioelectric dynamics provide stronger or more interpretable signals.

Future research should focus on mechanistic investigation through controlled physical measurements, distance-dependent studies to characterize signal propagation, enhanced temporal analysis with longer recording periods, multi-channel spatial analysis to preserve electrode-specific information, and replication studies across different facilities and plant populations. A critical next step is to add a “human present, non-gesture movement” condition and to monitor micro-airflow, VOC concentration, vibration, and electromagnetic fluctuations with appropriate sensors. Only with such measurements can one test whether subtle airflow or thermal boundary-layer perturbations, rather than plant-intrinsic bioelectric responses, drive the observed differences. Increasing recording duration beyond 3–8 s will also allow evaluation of slower physiological responses (e.g., variation potentials), which may be invisible in the short windows used here.

Taken together, these limitations underscore that the present results represent exploratory correlations rather than mechanistic evidence of plant sensing. Detectable differences were small, highly confounded, and derived from short windows and generic feature representations; controlled follow-up studies are required to establish whether true plant physiological responses are present.

## 5. Conclusions

This study provides exploratory evidence that plants exhibit modest but statistically detectable bioelectric differences when a human is present and moving nearby. While the current experimental design cannot isolate the specific contribution of eurythmic gestures from other proximal human influences (e.g., airflow, thermal, electromagnetic, or acoustic factors), the consistent classification performance across multiple methods (57–63%) suggests that genuine signal differences are present.

The study’s contributions are threefold: (1) demonstration that machine learning can extract above-chance distinctions between plant signals recorded with and without nearby human movement; (2) identification of performer-specific signatures in plant bioelectric recordings; and (3) preliminary evidence that repeated exposures may shift baseline bioelectric activity.

These results should be interpreted cautiously. Effect sizes are modest, the underlying mechanisms remain unresolved, and practical applications as plant-based biosensors are a long-term prospect. Nevertheless, this work establishes baseline metrics for plant–human bioelectric studies and motivates future research with tighter environmental controls, additional human movement conditions, and mechanistic investigation.

## Figures and Tables

**Table 1:** Two-sided Wilcoxon signed-rank test for paired “silent” pre- or post-gesture baseline recording and Eurythmy gesture recording.

Feature	N	Median (Baseline)	Median (Gesture)	*p*-Value
mfcc11	292	15.0704842	13.9413362	1.56 × 10^−16^
mfcc8	292	3.38241947	4.84369445	6.31 × 10^−16^
spec_centroid	292	0.5360596	0.72475345	1.27 × 10^−14^
wavelet_E5	292	0.00058634	0.00014236	3.25 × 10^−14^
spec_rolloff85	292	0.68359375	0.87890625	3.30 × 10^−14^
zcr	292	0.00250156	0.00375235	3.05 × 10^−13^
mfcc12	292	12.0499167	10.9974027	8.92 × 10^−10^
mfcc10	292	15.2547159	14.6643543	2.04 × 10^−9^
skew	292	−0.5198816	−0.2024614	2.26 × 10^−9^
mfcc5	292	19.1944084	17.8135567	8.28 × 10^−9^
mfcc9	292	10.8400011	11.6041932	8.28 × 10^−9^
mfcc3	292	86.1057701	91.359169	7.87 × 10^−8^

## Data Availability

The datasets generated and analyzed during the current study are available from figshare https://figshare.com/articles/dataset/Machine_Learning_Detection_of_Plant_Bioelectric_Responses_to_Human_Eurythmic_Gestures_/30227083?file=58324288 (accessed 25 October 2025).
